# Depressive Symptoms Are Associated with Mental Stress-Induced Myocardial Ischemia after Acute Myocardial Infarction

**DOI:** 10.1371/journal.pone.0102986

**Published:** 2014-07-25

**Authors:** Jingkai Wei, Pratik Pimple, Amit J. Shah, Cherie Rooks, J. Douglas Bremner, Jonathon A. Nye, Ijeoma Ibeanu, Nancy Murrah, Lucy Shallenberger, Paolo Raggi, Viola Vaccarino

**Affiliations:** 1 Department of Epidemiology, Rollins School of Public Health, Emory University, Atlanta, Georgia, United States of America; 2 Department of Medicine, Division of Cardiology, Emory University School of Medicine, Atlanta, Georgia, United States of America; 3 Department of Psychiatry and Behavioral Sciences, Emory University School of Medicine, Atlanta, Georgia, United States of America; 4 Department of Radiology, Emory University School of Medicine, Atlanta, Georgia, United States of America; 5 Mazankowski Alberta Heart Institute, University of Alberta School of Medicine, Edmonton, Alberta, Canada; Carleton University, Canada

## Abstract

**Objectives:**

Depression is an adverse prognostic factor after an acute myocardial infarction (MI), and an increased propensity toward emotionally-driven myocardial ischemia may play a role. We aimed to examine the association between depressive symptoms and mental stress-induced myocardial ischemia in young survivors of an MI.

**Methods:**

We studied 98 patients (49 women and 49 men) age 38–60 years who were hospitalized for acute MI in the previous 6 months. Patients underwent myocardial perfusion imaging at rest, after mental stress (speech task), and after exercise or pharmacological stress. A summed difference score (SDS), obtained with observer-independent software, was used to quantify myocardial ischemia under both stress conditions. The Beck Depression Inventory-II (BDI-II) was used to measure depressive symptoms, which were analyzed as overall score, and as separate somatic and cognitive depressive symptom scores.

**Results:**

There was a significant positive association between depressive symptoms and SDS with mental stress, denoting more ischemia. After adjustment for demographic and lifestyle factors, disease severity and medications, each incremental depressive symptom was associated with 0.14 points higher SDS. When somatic and cognitive depressive symptoms were examined separately, both somatic [β = 0.17, 95% CI: (0.04, 0.30), p = 0.01] and cognitive symptoms [β = 0.31, 95% CI: (0.07, 0.56), p = 0.01] were significantly associated with mental stress-induced ischemia. Depressive symptoms were not associated with ischemia induced by exercise or pharmacological stress.

**Conclusion:**

Among young post-MI patients, higher levels of both cognitive and somatic depressive symptoms are associated with a higher propensity to develop myocardial ischemia with mental stress, but not with physical (exercise or pharmacological) stress.

## Introduction

Depression is a prevalent, debilitating, and underdiagnosed condition that affects approximately 20% of patients with recent myocardial infarction (MI), and is associated with increased risk of recurrent cardiac events [1]. Recent literature suggests that specific depressive symptom dimensions carry different risks with respect to cardiovascular prognosis. Specifically, somatic symptoms of depression, such as sleep, appetite disturbance and fatigue, have been reported in several studies as being more predictive of cardiac events in patients with coronary artery disease (CAD) than cognitive symptoms, such as feelings of sadness, pessimism, and failure [2], [3].

The precise mechanisms through which depression may increase the risk for adverse cardiovascular outcomes after an acute MI are not known. Adverse behaviors, inflammation and abnormal autonomic function have been linked to both depression and recurrent CAD events, but whether these factors explain the increased risk associated with depression after MI is debatable [4]–[6]. One rarely considered possibility is that depressed post-MI patients are more likely to develop myocardial ischemia during psychological stress [7], [8]. Ischemia induced by psychological stress is a common phenomenon in stable CAD patients [9] and is associated with a doubling of risk of cardiac events and death [10]. This phenomenon can be studied experimentally in the laboratory by using a standardized mental stress test [11], but it has rarely been evaluated with respect to depression.

We systematically tested a sample of young and middle-aged post-MI patients, a group with high burden of psychosocial distress [12], [13], for inducible ischemia with both mental stress and exercise/pharmacological stress using single-photon emission computed tomography (SPECT). We addressed the hypothesis that an increased level of depressive symptoms is a risk factor for mental stress-induced ischemia, but not for exercise/pharmacological stress-induced ischemia. Furthermore, we examined whether somatic and cognitive symptoms of depression differed in their association with mental stress-induced ischemia.

## Methods

### Subjects

Participants for this study were recruited from the pool of patients younger than 60 years of age admitted in the previous 6 months with a confirmed diagnosis of MI at Emory-affiliated hospitals as part of the Myocardial Infarction and Mental Stress Study (MIMS). Details of our recruitment strategy and number of eligible and excluded patients have been described [14]. An equal proportion of men and women was recruited; men and women were matched by age, type of MI and time of enrollment since the index MI. Subjects were excluded if they had unstable angina or acute MI within the past week, or they had a severe comorbid medical or psychiatric disorder that could interfere with study results, such as cancer, renal failure, current alcohol or substance abuse or schizophrenia; if they weighed over 400 pounds, if they were pregnant or breastfeeding, or if they were currently using postmenopausal hormone therapy or psychotropic medications other than antidepressants. Patients were also excluded if they were unable to exercise on a treadmill, based on a score <5 metabolic equivalents (METs) on the Duke Activity Status Index (DASI), which identifies patients who cannot exercise to heart rate targets [15].

### Study Design

Study subjects underwent three SPECT imaging scans, one with rest, one with mental stress and one with physical stress. The latter included predominantly exercise stress. However, 16 patients were unable to reach their target heart rate despite scoring ≥5 METs on the DASI and therefore underwent a pharmacological stress test. The two stress scans were performed on separate days, mostly within one week of each other (on average, 4 days apart, median 5 days, interquartile range 3 days), and the rest scan was performed on the first visit. The order of the two types of stress tests was counterbalanced. All testing was done after an overnight fast, and anti-ischemic medications were held for 24 hours prior to testing. Sociodemographic and psychosocial data were collected at the first visit prior to cardiac testing. At the end of the study protocol, medical records were abstracted for clinical information, including catheterization data. All participants signed a written informed consent. The study protocol and informed consent were approved by the Emory University Institutional Review Board.

### Mental Stress Procedure

Initially, patients rested for 30 minutes in a quiet, dimly lit, temperature-controlled room. At the end of the resting period, mental stress was induced by a standardized public speaking task as previously described [16]. Patients were asked to imagine a real-life stressful situation, such as a close relative been mistreated in a nursing home, and asked to make up a realistic story around this scenario. They were given two minutes to prepare a statement and then three minutes to present it in front of a video camera and an audience wearing white coats. Subjects were told that their speech would be evaluated by the laboratory staff for content, quality and duration.

### Myocardial Perfusion Imaging

Subjects underwent three SPECT myocardial perfusion imaging scans following injection of sestamibi radiolabelled with Technetium-99m (^99m^Tc sestamibi), at rest, after mental stress, and after physical (exercise or pharmacological) stress. Testing was done on a dedicated ultra-fast solid-state camera (Discovery NM 530c, General Electric, Milwaukee, WI) without attenuation correction [17]. Only one resting scan was performed, with myocardial perfusion images acquired after the injection of 10–15 mCi of [^99m^Tc] sestamibi according to body weight. The stress scan (either mental or physical) followed at least 2 hours later and 30–45 mCi of [^99m^Tc] sestamibi were administered, with a 3∶1 ratio of stress to rest radiotracer dose.

On the mental stress day, [^99m^Tc]sestamibi was injected one minute after the onset of the public speech. On the exercise stress day, subjects were submitted to a standard Bruce protocol with exercise target set at 85% of maximum predicted heart rate and the radiotracer was injected at peak exertion. For both stress conditions, stress images were acquired 45–60 minutes after radiotracer injection using previously described methodology [18]. The ECG, blood pressure and heart rate were continuously monitored during the test. For patients undergoing a pharmacological stress test, 0.4 mg of regadenoson (Astellas, Northbrook, Illinois), an adenosine receptor agonist, were administered intravenously in approximately 10 seconds, and [^99m^Tc] sestamibi was injected right after regadenoson. SPECT images were then obtained as described above.

Myocardial perfusion abnormalities were quantified by means of the Emory Cardiac Toolbox software, which provides objective quantitative assessment of perfusion with established validity and reproducibility [19], [20]. Automated analysis for SPECT myocardial perfusion imaging is equivalent to visual analysis by expert readers [21], [22], but is more reproducible, since interpreter variability is an important source of heterogeneity in imaging results [23], [24]. With automated analysis, such variability is removed, making this technique more reproducible and thus better suited for studies with serial SPECT scans such as ours. Briefly, the three-dimensional tracer uptake distribution in the left ventricle was oriented along the short axis and sampled onto a two-dimensional polar map. An operator-independent summed score, quantifying the extent and severity of perfusion defects across 17 segments of the myocardium at rest and during stress, was computed by the software according to published methodology [20]. The regional severity scoring was then summed up across the 17 myocardial segments yielding a total score. Separate scores were obtained for the rest images (summed rest score, SRS) and the stress images (summed stress score, SSS). A summed difference score (SDS), quantifying the number and severity of reversible (ischemic) myocardial perfusion defects, was obtained by subtracting the rest score from the stress score; in the presence of a reversible defect (or ischemia), the score is positive. In secondary analyses, regional severity scoring was calculated according to vascular territories designated by standard techniques, i.e., left anterior descending (LAD), left circumflex (LCx), and right coronary artery (RCA).

### Measurements of Depressive Symptoms

Depressive symptoms were assessed with the Beck Depression Inventory-II (BDI-II) [25], a reliable and valid self-administered questionnaire which has been widely used in cardiac populations. The BDI-II includes 21 items, each containing 4 statements scored 0 to 3, with higher scores indicating higher severity of depression. Two separate depressive symptoms scores were constructed according to symptom dimensions, following established methods [25]. Somatic symptoms included items such as sleep, fatigability and appetite, and cognitive symptoms included items such as sadness, guilty feelings, pessimism and sense of failure.

### Other Measurements

Information on sociodemographic factors was collected using standard questionnaires from population studies. A detailed medical history, including medication use, was obtained by a research nurse. Weight and height were used to calculate body mass index. Venous blood samples were drawn for the measurements of glucose and lipid profile after an overnight fast.

Angiographic data were obtained from the coronary angiogram performed in conjunction with the index MI. CAD severity was quantified using the Gensini semi-quantitative angiographic scoring system [26], which takes into account degree of luminal narrowing along with a multiplier for specific coronary tree locations. This score was corrected according to the revascularization procedure results, i.e., the percentage of coronary obstruction used in the score computation reflected the post-revascularization vessel status. From coronary angiograms or echocardiography reports, we also abstracted the left ventricular ejection fraction.

### Statistical Analysis

Descriptive statistics were computed by comparing mean BDI-II scores according to levels of other study variables. Next, multivariate linear regression models were used to examine the association between depressive symptoms and stress perfusion scores adjusting for possible confounding factors. The SDS, which quantifies ischemia, was our main outcome of interest. Since the SDS for both mental and physical stress was highly skewed, while the SSS was approximately normally distributed, we used the SSS scores as dependent variables while adjusting for the rest score (SRS), yielding coefficients for depression identical to those from a model where the dependent variable is the difference score (SDS), adjusted for the SRS. In a series of cumulative hierarchical models, we adjusted for a set of factors that were considered a-priori as either possible confounding factors or mediators of the relationships under study. Adjustment factors included sociodemographic and lifestyle characteristics (sex, employment, race, marital status and cigarette smoking), medications (use of statins, beta-blockers and anti-depressants), CAD severity (Gensini score, left ventricular ejection fraction), traditional CAD risk factors (history of diabetes and hypertension, and BMI), and previous revascularization procedures (coronary artery bypass grafting and percutaneous coronary intervention).

## Results

### Study Sample

Between 2009 and 2012, 49 male and 49 female MI patients younger than 60 years were included in the study. The mean and median age was 50 years. Overall, 55% of the participants were African-American; 57% had at least high school education and 32% had an income below the poverty level (**Table 1**). Forty-five percent of the patients had an ST-elevation MI, 74% had previous percutaneous coronary interventions, and 20% had previous coronary artery bypass graft surgery.

**Table 1:** Mean depressive symptoms (BDI-II total score) according to patient characteristics.

	N (%)	BDI-II Total Score Mean (SD)	p value
Sex			
Male	49 (50%)	9.4 (6.8)	0.042
Female	49 (50%)	13.0 (9.8)	
Age			
≤50	49 (50%)	10.9 (8.6)	0.718
>50	49 (50%)	11.5 (8.7)	
Race			
Black	54 (55.1%)	10.4 (9.3)	0.285
Non-Black	44 (44.9%)	12.2 (7.7)	
Currently Married			
Yes	40 (40.8%)	10.6 (7.0)	0.551
No	58 (59.2%)	11.6 (9.6)	
Education			
High school or more	56 (57.1%)	10.1 (7.5)	0.130
Less than high school	42 (42.9%)	12.7 (9.8)	
Employment status			
Employed	81 (82.7%)	13.2 (10.1)	0.298
Unemployed	17 (17.3%)	10.8 (8.3)	
Income below poverty level			
Yes	31 (31.6%)	14.5 (9.8)	0.011
No	65 (66.3%)	9.3 (6.9)	
Current smoking			
Yes	28 (28.6%)	14.9 (10.2)	0.021
No	70 (71.4%)	9.7 (7.4)	
Hypertension			
Yes	67 (68.4%)	12.2 (8.6)	0.106
No	30 (30.6%)	9.2 (8.3)	
Diabetes			
Yes	20 (20.4%)	11.7 (10.9)	0.807
No	77 (78.6%)	11.2 (8.0)	
Obesity			
Yes	45 (45.9%)	12.0 (8.9)	0.445
No	52 (53.1%)	10.7 (8.4)	
MI Type			
ST-Elevation MI	44 (44.9%)	10.7 (8.0)	0.61
Non ST-Elevation MI	54 (55.1%)	11.6 (9.1)	
Previous Revascularization Procedures			
CABG			
Yes	20 (20.4%)	10.1 (10.0)	0.51
No	76 (77.6%)	11.5 (8.2)	
PCI			
Yes	73 (74.5%)	11.6 (8.6)	0.45
No	21 (21.4%)	10.0 (9.2)	
Current Medications			
Statins			
Yes	85 (86.7%)	13.2 (10.2)	0.610
No	12 (12.2%)	9.6 (7.6)	
Beta blockers			
Yes	85 (86.7%)	11.9 (8.7)	0.071
No	12 (12.2%)	7.1 (7.3)	
ACE-Inhibitors			
Yes	53 (54.1%)	12.5 (9.2)	0.129
No	44 (44.9%)	9.8 (7.7)	
Aspirin			
Yes	85 (86.7%)	11.2 (8.7)	0.705
No	12 (12.2%)	12.2 (8.0)	
Anti-depressants			
Yes	13 (13.3%)	21.4 (8.8)	<0.001
No	84 (85.7%)	9.7 (7.5)	

Abbreviations: BDI-II: Beck Depression Inventory-II; SD: standard deviation; ACE-Inhibitors: angiotensin-converting-enzyme inhibitors; CABG: coronary artery bypass graft; PCI: percutaneous coronary intervention; MI: myocardial infarction.

### BDI-II Scores

The BDI-II total scores were approximately normally distributed, with a mean of 11.2 (SD: 8.6, range: 0–37). The mean somatic symptom score was 2.8 (SD: 3.3, range: 0–13), and the mean cognitive symptom score was 8.4 (SD: 6.1, range: 0–24). BDI-II total scores were higher among women, among patients with income below poverty and among current smokers (**Table 1**).

### Myocardial Perfusion

Three patients, 1 woman and 2 men, had missing myocardial perfusion data for both mental stress and exercise or pharmacological stress, and were excluded from the perfusion imaging analyses. The median SDS with mental stress was 2.0 [interquartile range (IQR): 0, 3], and the median SDS with physical stress was also 2.0 (IQR: 0, 5). The SDS with mental stress and physical stress were not correlated (Spearman r = 0.11, p = 0.31). Results were similar with no significant heterogeneity based on whether exercise stress or pharmacological stress was used.

### Association of Depressive Symptoms with Myocardial Ischemia Severity

**Figure 1:**
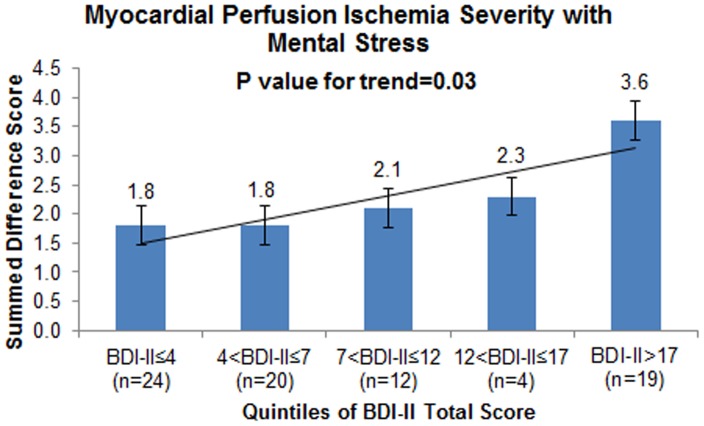
Mean unadjusted myocardial perfusion ischemic defect severity [raw summed difference score (SDS)] with mental stress according to five groups of progressively higher depressive symptoms using quintiles of the BDI-II total score. The error bars represent standard errors. The p-value is from a linear regression model where quintiles of the BDI-II score were modeled as an ordinal variable. There was a statistically significant progressive increase in mental stress-induced myocardial ischemia with increasing depressive symptom severity.

**Figure 2:**
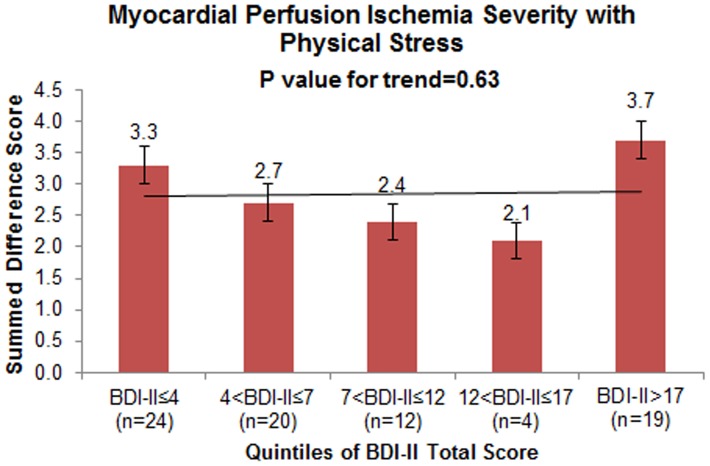
Mean unadjusted myocardial perfusion ischemic defect severity [raw summed difference score (SDS)] with physical (exercise or pharmacological) stress according to five groups of progressively higher depressive symptoms using quintiles of the BDI-II total score. The error bars represented standard errors. The p-value is from a linear regression model where quintiles of the BDI-II score were modeled as an ordinal variable. There was no statistical difference in physical stress-induced myocardial ischemia with increasing depressive symptom severity. No association was also found when non-parametric regression with smoothing splines was used to model a non-linear association.

A higher BDI-II total score was associated with increased propensity for ischemia with mental stress. The graded fashion of this association was evident when the BDI-II total score was categorized according to quintiles (**Figure 1**). After adjustment for demographic and lifestyle factors, CAD severity and medications, each 1-point increase in BDI-II total score was associated with 0.14 points increase in SDS with mental stress (95% CI: 0.03 to 0.24, p = 0.01). In contrast, the BDI-II total score was not associated with the SDS with physical stress (**Figures 2** and **Table 2**).

**Table 2:** Association between depressive symptoms (BDI-II total score) and myocardial ischemia severity, as quantified by the SDS with mental stress and with physical (exercise or pharmacological) stress.

	β (95% CI)†	P value
**Mental Stress**		
Model 1: Unadjusted	0.07 (0.01, 0.14)	0.02
Model 2: Adjusted for demographic and lifestyle factors‡	0.07 (0.01, 0.14)	0.04
Model 3: Adjusted for the above plus CAD severity, traditional risk factors and medications§	0.14 (0.05, 0.23)	0.01
**Physical Stress**		
Model 1: Unadjusted	−0.01 (−0.09, 0.07)	0.75
Model 2: Adjusted for demographic and lifestyle factors‡	−0.01 (−0.09, 0.08)	0.73
Model 3: Adjusted for the above plus CAD severity, traditional CAD risk factors and medications§	0.06 (−0.05, 0.17)	0.29

Abbreviations: BDI-II: Beck Depression Inventory-II; SDS: summed difference score; CI: confidence interval; CAD: coronary artery disease.

† The β coefficient expresses the difference in SDS score points with a 1-point increase in BDI-II total score. Each model was constructed with SSS as dependent variable adjusting for the rest score (SRS). SE: standard error.

‡ Sex, employment, race, marital status and cigarette smoking.

§ Gensini angiographic CAD severity score, left ventricular ejection fraction, hypertension, diabetes, BMI, previous revascularization procedures, use of statins, beta-blockers, and anti-depressants.

Next, depressive symptoms were analyzed as separate somatic and cognitive components (**Table 3**). Somatic depressive symptoms were associated with ischemic perfusion defect severity with mental stress in both unadjusted and adjusted analysis. In the final model there was a 0.17-point increase in SDS for each increment in somatic symptoms (β = 0.17, 95% CI, 0.04 to 0.30, p = 0.01). For cognitive depressive symptoms, the unadjusted association was marginally significant, but it strengthened in the final adjusted model (β = 0.31, 95% CI, 0.07 to 0.56, p = 0.01). Neither symptom dimension was associated with physical stress ischemia.

**Table 3:** Association between BDI-II somatic and cognitive symptom scores and myocardial ischemia severity with mental stress, as quantified by the SDS.

	β (95% CI)†	P value
**Somatic Depressive Symptoms**		
Model 1: Unadjusted	0.10 (0.01, 0.19)	0.03
Model 2: Adjusted for demographic and lifestyle factors‡	0.10 (0.0003, 0.20)	0.05
Model 3: Adjusted for the above plus CAD severity, traditional risk factors and medications§	0.17 (0.04, 0.30)	0.01
**Cognitive Depressive Symptoms**		
Model 1: Unadjusted	0.16 (−0.01, 0.33)	0.06
Model 2: Adjusted for demographic and lifestyle factors‡	0.16 (−0.01, 0.34)	0.15
Model 3: Adjusted for the above plus CAD severity, traditional risk factors and medications§	0.31 (0.07, 0.56)	0.01

Abbreviations: BDI-II: Beck Depression Inventory-II; SDS: summed difference score; CI: confidence interval; CAD: coronary artery disease.

† The β coefficient expresses the difference in SDS score points with a 1-point increase in BDI-II total score. Each model was constructed with SSS as dependent variable adjusting for the rest score (SRS). SE: standard error.

‡ Sex, employment, race, marital status and cigarette smoking.

§ Gensini angiographic CAD severity score, left ventricular ejection fraction, hypertension, diabetes, BMI, previous revascularization procedures, use of statins, beta-blockers, and anti-depressants.

There were no significant differences in results according to sex, although the association between depressive symptoms and mental stress-induced ischemia appeared stronger among men. To rule out a possible nonlinear trend in the association between BDI-II and physical stress ischemia, we repeated the analysis using non-parametric regression using smoothing splines [27], and again found no association (p = 0.90). Analysis based on vascular territories was consistent with the overall results. Under mental stress, there was an association of roughly similar magnitude between BDI-II and SDS in the LAD territory (β = 0.034, p = 0.07) and in the LCx territory (β = 0.039, p = 0.04), but not in the RCA territory (β = 0.008, p = 0.62). In contrast, in none of the vascular territories there was an association between BDI-II score and SDS with physical stress.

## Discussion

In this experimental study of young and middle-aged post-MI patients, we demonstrated a robust association between baseline depressive symptoms and inducible myocardial ischemia with mental stress, but not with exercise/pharmacological stress. We observed this association for both somatic and cognitive depressive symptoms. Given that mental stress-induced ischemia is a prognostic indicator in CAD patients [10], these findings provide a possible explanation for the increased risk of adverse events associated with depression in post-MI patients [28], [29].

Only a small number of studies have examined the association between depressive symptoms and mental stress-induced myocardial ischemia, yielding inconsistent results. Jiang et al. [30] found a significant association between depressive symptoms measured by the Center for Epidemiological Studies-Depression Scale and the probability of mental stress-induced ischemia in stable CAD patients. Similarly, Boyle et al. [31] found a significant association between the BDI-II score and inducible ischemia with mental stress; these authors also found that somatic depressive symptoms were better predictors of mental stress-induced ischemia than cognitive symptoms. On the other hand, Ketterer et al. [11] and Burg et al. [32] did not detect a significant association between depression and mental stress-induced myocardial ischemia in patients with CAD. These discrepancies may be due to variations in study populations and research protocols. These previous investigations included predominantly older, male and Caucasian patients, and used radionuclide ventriculography or echocardiography to assess ischemia. In contrast, we examined young and middle-age patients with a large proportion of minority participants and an equal representation of men and women. Additionally, we focused on a well-characterized post-MI population within 6 months of their index MI, while all previous investigators examined patients with different medical histories and disease stages. Finally, we used [^99m^Tc]sestamibi SPECT myocardial perfusion imaging to detect ischemia, which is a customary method for the detection of ischemia in contemporary clinical care.

Potential mechanisms that might be invoked to explain the association between depressive symptoms and mental stress-induced myocardial ischemia include downstream physiologic effects of stress-induced activation of the hypothalamic-pituitary-adrenal (HPA) axis, which can be dysregulated in depression [33]. Furthermore, inflammation, which is enhanced in depression, may be implicated. Patients with CAD display increased inflammatory responses to mental stress [34], and inflammation is associated with abnormal coronary microvascular function [35], which, in turn, could be a mechanism of mental stress-induced ischemia [36]. Autonomic nervous system dysfunction is also a common factor in both depression and CAD [4]. Previous studies have shown that the autonomic nervous system, especially parasympathetic withdrawal, plays a significant role in the pathophysiology of ambulatory ischemic episodes and may be responsible for triggering ischemia [37].

In our study, depressive symptoms were associated only with mental stress-induced myocardial ischemia and not with physical stress-induced ischemia. This finding differs from the study by Boyle et al. [31], in which depressive symptoms were associated with both mental stress and exercise-induced myocardial ischemia. Again, these discrepancies may be due to differences in study populations. Our findings imply that, among younger post-MI patients, mental stress- and physical stress-induced ischemia are distinct phenomena with unique triggers. Our results are corroborated by emerging evidence that mental stress- and physical stress-induced ischemia have different pathophysiological substrates. Mental stress-induced ischemia likely involves microvascular dysfunction, resulting in abnormal coronary or peripheral vasomotion due to enhanced vasoconstrictive responses to mental stress. In contrast, physical stress-induced ischemia is induced primarily by coronary steal occurring when restricted vasodilation in diseased epicardial vessels causes selective hypoperfusion [36], [38].

Our findings have substantial relevance for public health and clinical medicine. Depression affects approximately 20% of post-MI men and 40% of post-MI women below the age of 60 [13], many of whom may experience ischemia with psychological stress in everyday life. Unlike physical stress-induced ischemia, ischemia triggered by mental stress is mostly silent and not associated with electrocardiographic changes [38], thus it may go unrecognized. Recently, the REMIT trial found that a 6-week regimen with escitalopram resulted in a lower rate of mental stress-induced ischemia in stable CAD patients compared with a placebo group. Thus, pharmacological treatment of depression could be a promising strategy to reduce mental stress-induced ischemia [39]. It is not yet known, however, whether reducing the frequency of mental stress ischemia through pharmacological interventions or other methods translates into better clinical outcomes. It is also unknown if such interventions, which have been tested in broadly selected populations of CAD patients, are equally useful in young post-MI patients who suffer from disproportionately high rates of depression.

The main limitation of our study is its small sample size, which may decrease statistical power. In addition, given that the study was restricted to young post-MI patients, our results may not be generalizable to other stable CAD patient populations or older age groups. However, we targeted an equal representation of women and men, and did not restrict study participation based on demographic factors other than age and sex. The other sociodemographic characteristics of our sample (high proportion of minorities, low socioeconomic status) are likely a result of the relatively young age and high proportion of women in our sample. Another limitation is lack of data linking mental stress ischemia to clinical outcomes. Nonetheless, a strength of our study is the use of a standardized mental stress protocol and a well-defined, relatively understudied patient population with an elevated prevalence of depressive symptoms. Furthermore, we used [^99m^Tc]sestamibi SPECT for the detection of myocardial ischemia, which has established sensitivity, specificity, and reproducibility to detect mental stress-induced myocardial ischemia [40]. An important advantage of this method is that [^99m^Tc]sestamibi, once injected during mental stress, is trapped in the myocyte, thus providing a real-time “snapshot” of perfusion at the time of stress. It is likely that this feature enhances the accuracy of ischemia assessment during mental stress.

In conclusion, depressive symptoms are associated with mental stress-induced myocardial ischemia in younger post MI patients. This association is robust even after multivariate adjustments for other risk factors and disease severity, and applies to both somatic and cognitive depressive symptoms. Since CAD patients who experience ischemia with mental stress are at elevated risk for new cardiac events and death [10], this phenomenon could help explain the adverse outcomes associated with depression in post-MI patients. Our findings may provide novel avenues for risk prediction, management and secondary prevention in this high-risk patient population.
